# Are There Knots in Chromosomes?

**DOI:** 10.3390/polym9080317

**Published:** 2017-08-02

**Authors:** Jonathan T. Siebert, Alexey N. Kivel, Liam P. Atkinson, Tim J. Stevens, Ernest D. Laue, Peter Virnau

**Affiliations:** 1 Department of Physics, Johannes Gutenberg University Mainz, Staudinger Weg 9, 55128 Mainz, Germany; jsiebert@uni-mainz.de (J.T.S.); akivel@students.uni-mainz.de (A.N.K.); 2 Department of Biochemistry, University of Cambridge, 80 Tennis Court Road, Cambridge CB2 1GA, UK; liamatkinson@gmail.com (L.P.A.); e.d.laue@bioc.cam.ac.uk (E.D.L.); 3 MRC Laboratory of Molecular Biology, Francis Crick Avenue, Cambridge Biomedical Campus, Cambridge CB2 0QH, UK; tjs23@cam.ac.uk

**Keywords:** knots, chromosomes, chromosome territories, DNA, fractal globule

## Abstract

Recent developments have for the first time allowed the determination of three-dimensional structures of individual chromosomes and genomes in nuclei of single haploid mouse embryonic stem (ES) cells based on Hi–C chromosome conformation contact data. Although these first structures have a relatively low resolution, they provide the first experimental data that can be used to study chromosome and intact genome folding. Here we further analyze these structures and provide the first evidence that G1 phase chromosomes are knotted, consistent with the fact that plots of contact probability vs sequence separation show a power law dependence that is intermediate between that of a fractal globule and an equilibrium structure.

## 1. Introduction

**Figure 1:**
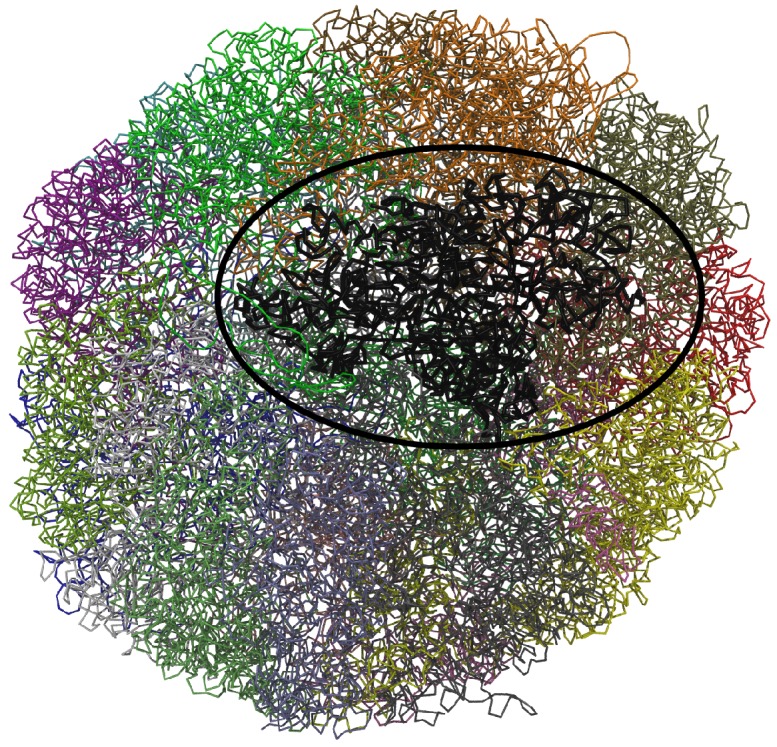
Structure of the intact genome of model 3 from cell No. 2. Chromosomes are shown in different colors [49]. Chromosome 14 (colored black), which is studied in this paper, is shown with thicker lines and is highlighted using a black ellipse.

Although very significant advances have been made in the last decades, understanding how chromosomes are organized in the cell nucleus remains a grand challenge in molecular biology [1,2,3,4,5,6,7,8,9,10,11,12]. During cell division, the segregation of daughter chromosomes requires the extensive reorganization of chromatin fibers from an unfolded state to a compact cylindrical shape. After cell division chromosomes then subsequently unfold again during early G1 phase to form chromosome territories [13], where each chromosome occupies a localized, largely non-overlapping region within the nucleus (see Figure 1). Current models suggest that chromosome compaction during mitosis involves Condensin complexes, which are thought to form rings that encircle two chromatin fibers. These Condensin mediated rings replace and extend links formed by the structurally similar Cohesin complexes, in a process that is assisted by Topoisomerase II (for a recent review see [14]). During mitotic chromosome condensation Topoisomerase II catalyzes double strand breaks in one DNA helix allowing the passage of a second helix through the break site, and plays a critical role in altering DNA topology [15]. Importantly, inhibition of this enzyme results in the formation of extended and unresolved metaphase chromosomes [16]. Topoisomerase II also plays a crucial role in releasing torsional stress in processes such as RNA transcription and DNA replication during interphase. For example, converging DNA replication forks cause a build-up of torsional stress when they approach each other, swivelling of the replication complexes, intertwining of newly replicated DNA molecules behind the forks, and the formation of catenanes. During mitosis, inter-chromosomal, but not intra-chromosomal, crossovers are thought to be identified and subsequently removed by Topoisomerase II (for a review see [17]).

To appreciate the difficulty of organizing these profound changes in chromatin structure one needs to consider the scales that are involved. DNA is a molecule with a diameter of about 2 nm. The total length of DNA in a single eukaryotic cell is on the order of a meter, which needs to be stored in a nucleus the diameter of which is around 10 micrometers. As suggested in [18], it is instructive to scale up these dimensions. If we increase all sizes by 1000, the problem of organizing DNA in the nucleus would then amount to storing 1 km of thread, cut into 20 pieces each 100 times thinner than a human hair, into a marble. It is hard to imagine how the rapid changes in chromosome structure, that occur in just a few hours of each cell cycle, might actually be possible without forming entanglements or knots.

From daily experience we know that cables and strings tend to form knots in confined environments. Computer simulations suggest that this also applies on the microscale if globular or confined polymers or DNA are given enough time to equilibrate [19,20,21,22]. While proteins are mostly unknotted, apart from a few exceptions [23,24,25,26,27,28,29,30,31,32], viral DNA in bacteriophages has been shown to be highly knotted, at least in mutants in which both sticky ends are allowed to enter the capsid [33,34,35,36,37,38,39]. Proteins and viral DNA are also two examples where entanglements or the lack thereof emerge in biomolecular systems in a non-equilibrium context. While proteins may adopt non-degenerate ground states in the language of statistical physics, DNA in the bacteriophages investigated is fed through a loading channel by a motor, which leads to rather dense toroidal structures. DNA knots can also occur in equilibrium systems. Using gel-electrophoresis experiments, knotting probabilities for sequences of up to 10,000 DNA base pairs have been determined in the early 1990s [40,41]. Recently, experiments and simulations have extended this analysis to sequences of up to 500,000 base pairs. At these length scales, unconstrained DNA tends to be highly knotted [42,43].

**Figure 2:**
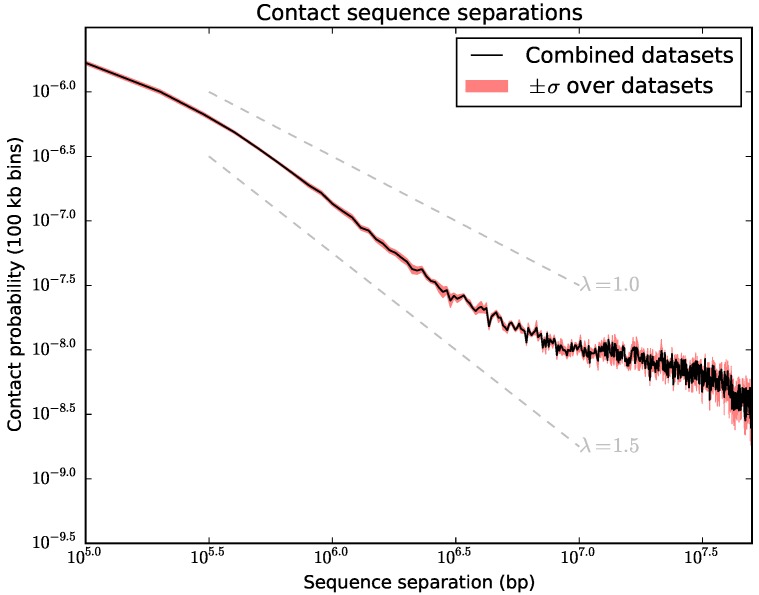
Variation in experimental contact probability with sequence separation calculated over all 8 cells. For equilibrium systems a power law decay ($P\left(s\right)\propto s^{-\lambda }$) of the contact probabilities with exponent λ=1.5 is expected, while the fractal globule model predicts λ=1.0 [46,47]. The exponent of the actual experimental data is in between λ=1.0 and λ=1.5 [48].

Chromosomes clearly belong to the class of non-equilibrium systems because typical equilibration times by far exceed the time spent in the different stages of the cell cycle—some theoretical estimates suggest that equilibration would take tens, if not hundreds of years [44]. This means that they will typically not reach a fully knotted equilibrium state in which all time and length scales are relaxed. For this reason, it has been suggested that the organization of chromosomes might better be described in terms of a so-called “fractal globule”. This concept is borrowed from polymer physics and describes a long-lived intermediate state emerging from the initial collapse of a polymer. Such a state is expected to be (mostly) unknotted as it doesn’t have enough time to relax its topology. The fractal globule is also consistent with the scaling laws observed for contact probability vs sequence separation for chromosomes in Hi–C data from populations of cells [18,45,46,47]. However, plots of contact probability vs sequence separation for single cells suggest a state that is intermediate between a fractal globule and an equilibrium system [48] (see Figure 2), implying that the structures may retain knots that were made during the preceding cell cycle(s). In this manuscript we test this hypothesis by analyzing the structures of intact single haploid mouse ES cell chromosomes with respect to knots.

## 2. Methods

**Figure 3:**
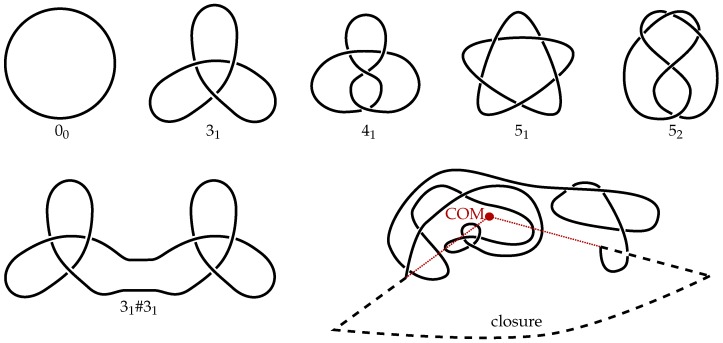
Schematic drawings of the five simplest knots ($0_{0}$, $3_{1}$, $4_{1}$, $5_{1}$, $5_{2}$) and an example of a composite knot ($3_{1}\#3_{1}$, containing two $3_{1}$ knots). On the lower right, the closure mechanism is drawn schematically. The ends of the chains are projected outward from the center of mass (COM). The chain is then closed far away from the chain by a large arc.

Mathematically, knots are only well-defined in closed curves and are characterized by the minimum number of crossings in a projection onto a plane. The simplest (non-trivial) knots are the so-called trefoil knot ($3_{1}$), which has the minimal three crossings, and the figure-of-eight knot ($4_{1}$) has four. Composite knots contain multiple prime knots, e.g., $3_{1}\#3_{1}$ is made up of two trefoil knots. These knots are shown in Figure 3 schematically. On closed curves, knots can be identified by use of knot polynomials. To close the chains, we apply the algorithm described in [27]. As shown in Figure 3, two lines are drawn projecting outwards from the center of mass, starting at the ends of the chain. By joining these two lines by a big loop, the chain is closed. Consecutively, knot types are determined by use of the Alexander polynomial. As the Alexander polynomial Δ(t) is undetermined by a factor of $\pm t^{m}$, we use the modified invariant Δp(t)=|Δ(−1.1)Δ(−1/1.1)| as proposed in [20].

Our results were generated by analyzing the 3D structures of the individual genomes of mouse haploid embryonic stem cells calculated previously [48]. These structures were generated based on experimental measurements of contact matrices determined by first imaging and then processing single cells. Using these contacts as constraints, the structures were first calculated at low resolution using simulated annealing of a particle on a string polymer model starting from different random conformations. These low-resolution structures were then used as independent starting configurations for more fine-grained simulated annealing calculations in a hierarchical scheme until the highest resolution was reached (where individual particles each represent 100,000 base pairs of DNA). The annealing procedure was started with 20 models at the lowest resolution. At an intermediate resolution of 400,000 base pairs per bead, half of the models were discarded and the computation was continued with 10 models. Details can be found in the supplementary information of [48]. For all the additional structures that were calculated for this paper, no selection was made at the intermediate resolution. In addition to the contact potential, there are additional potentials to keep sequentially adjacent particles in the chromosomes together, as well as a volume exclusion term to stop them overlapping. (The specific functional forms and parameters can be found in the supplementary information of [48] and in the code that was used, which is referenced therein.) It is important to note that the volume exclusion term is rather small and is turned on only during the cooling phase of the simulated annealing. Therefore, particles can pass through each other more or less freely at high temperatures. This ensures that the chain does not get kinetically trapped in unfavorable configurations, but it might introduce spurious knots in the structures due to the very simplified representation of the chromosome structure.

## 3. Results

**Figure 4:**
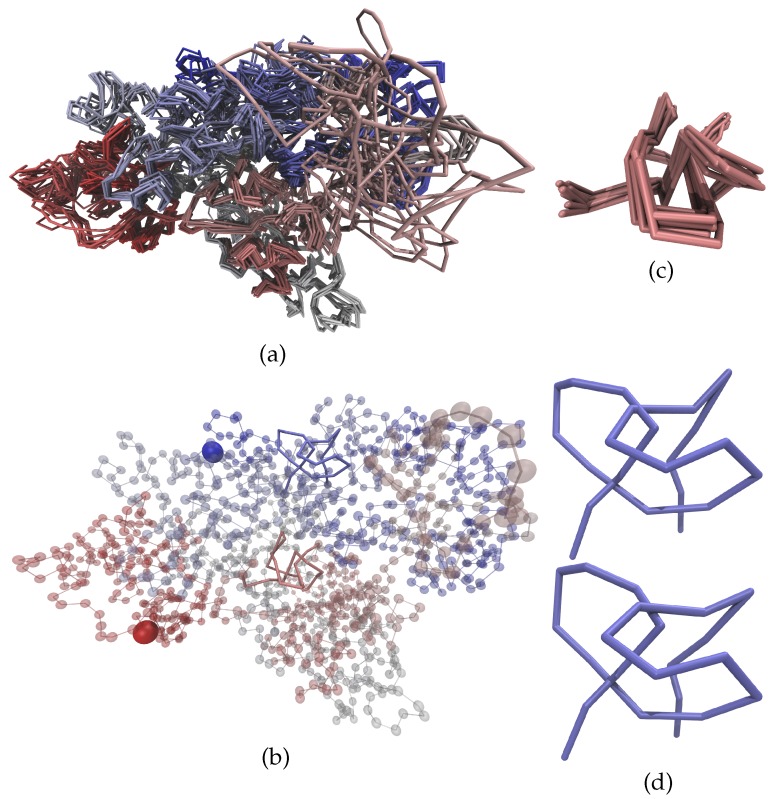
Structure of chromosome 14 from cell 2 [48]. Panel (**a**) shows a comparison of all 10 models of the complete chromosome; whilst panel (**b**) shows one of those models (model 3), which contains two trefoil knots; Panel (**c**) shows an expanded view of one of the regions that forms a knot. Generally, the different models show a remarkable consistency. This corroborates our belief that the experimentally measured contacts define a non-degenerate ground state that is properly determined by the annealing procedure [48]. However, it is worth noting that there are also loops of the chromosome at the nuclear surface that do not exhibit many contacts. One example is the salmon colored loop in the top right of panel (**a**). Due to a lack of contacts this loop is not in a well-defined ground state but differs rather strongly between different models. In contrast, the two regions discussed in this paper do exhibit a well-defined structure. In panel (**b**), model 3 is represented as transparent beads with the knotted regions highlighted by solid lines—termini are accentuated by large solid beads. The red knot as shown in panel (**c**) has a very well-defined structure in all the models. In combination with the comparably large separations between the arcs of the knots, this leads to a consistent topology for all models. The structure of the blue region shown in panel (**d**) differs more strongly between the different models. In the upper center of the structure the separation between two of the arcs is very small. This leads to differences in the topology for different models as the trefoil knot that is present in some of the models (e.g., in the upper structure) can easily vanish (as shown in the lower structure) when the two arcs exhibit only a small relative change in their positions.

In the calculated structures [48] chromosomes are characterized as linear open chains with between 582 and 1925 particles (or beads), where each bead corresponds to 100,000 base pairs. 10 different simulated annealing models were calculated independently (for eight individual cells) starting from different random conformations in which beads are placed randomly within a sphere. Remarkably, all the models for each individual cell showed very similar structures that only differ in a few regions in the vicinity of the nuclear envelope (where there are fewer experimental constraints) even though they emerge from completely independent starting conformations. This indicates that there is indeed something akin to a non-degenerate ground state defined by the experimental contacts, which can be found by the annealing procedure. The similarity of the different models to each other is shown in Figure 4 for chromosome 14 of cell 2 which is the example that will be illustrated throughout this manuscript. Although most of the structure is very similar for all the models, regions on the surface of the genome do vary considerably. One example is the salmon colored region in the upper right of panel (a). This can be easily understood because it represents an unmappable sequence of the genome and thus lacks experimental contacts with other parts of the same chromosome as well as with surrounding chromosomes. Nonetheless, the structures of the other parts of the chromosome are very well determined and do not vary strongly between the different models. This corroborates our belief that while there might be some topological artifacts arising from the simplified chromosome model and uncertainties in the calculation, the structures seem to be well determined.

We analyzed the topology of all the structures, closing the chains and computing their Alexander polynomial as described in [27]. Our results indicate that most of the chromosomes do contain knots, with the fraction of unknotted chromosomes being less than 20% (see Table 1). We found that the knots are rather complex—more than half of all chromosomes contain knots with more than five crossings or even multiple knots. We obtained similar results for knotting when the annealing protocol was modified to include an enhanced excluded volume potential (while in the original annealing procedure, the excluded volume interactions were only slowly turned on, now the full potential was used throughout the complete annealing protocol)—to reduce strand crossing during the structure calculation procedure. This involved testing the enhanced excluded volume potential with different starting conformations. From a random starting conformation, we were not able to compute structures directly at 100 kb resolution: the chromosomes were tangled and showed significant violations of the experimental restraints. However, we were able to compute low-resolution structures, from a random starting point, which were both unknotted and convergent—meaning that they reached a coherent structure (at this low resolution: RMSD<0.25beadradii) consistent with the experimental constraints. Then, when we used these low-resolution conformations as untangled starting structures for high-resolution calculations (at 100 kb and also with an enhanced excluded volume potential and lower annealing temperatures, starting at 300 K reducing to 10 K,) we obtained convergent (at the highest resolution: RMSD<1beadradius) knotted structures comparable to the original dataset. These knots arise because a stronger volume exclusion of the beads does not completely suppress strand crossing for sufficiently stretched bond lengths as the structures compact. Greatly increasing the bond potential remedies this, but it also results in distorted non-convergent structures. Furthermore, this suggests that a rigid bond length, for sequential 100 kb particles, is inconsistent with the experimental data.

We next generated unknotted structures from the published models by small random displacements of individual beads that were accepted only if the knot type became simpler. A re-equilibration of these conformations with the enhanced excluded volume potential also led to mostly knotted conformations (which differ slightly from the original structures due to the altered model potentials). All these tests indicate that the knotted (ground-state-like) structure is indeed defined by the underlying contact data and the computational model for a wide range of parameters.

An example of a knotted structure (Cell No. 2, Chromosome 14, Model 3) is shown in Figure 4 panel (b), where our analysis located two trefoil knots that are highlighted in red and blue. Our usual method to determine knot positions [20] typically overestimated the knot sizes significantly due to the density of the structures. Therefore, all knot positions and sizes reported in this manuscript had to be determined manually. For better visibility of the knotted structure, panels (c) and (d) show the red and blue knots on their own. The two knots occur in the middle of the chain and are unaffected by its closure. While the red knot, located between beads 301 and 329, is present in all models of the genome structure from this cell, the blue knot between beads 964 and 985 vanishes in some models due to a change in backbone crossing which alters the topology. This is visualized in Figure 4 panels (c) and (d) respectively. The structure of the red trefoil knot in panel c) mainly differs in overall position while the relative positions of the nodes seem to be fixed. Even though we did not analyze knot sizes in detail, we need to keep in mind that each bead in the model represents a hundred kilobases of DNA, whose detailed topology is not determined in our structures. The fact that this chromosome knot forms at the megabase level suggests, however, that our analysis may not be affected by the low resolution. The separation between the arcs of the knot is rather large such that even small changes in the relative positions of the nodes do not change the topology. In contrast, the structure of the blue region in panel (d) differs more strongly between the different models and is only knotted in some of the models. This is because of the closeness of the two arcs in the upper center of the blue region such that a slight change in the relative position of the nodes can cause a change in backbone crossing resulting in a changed knot type.

**Figure 5:**
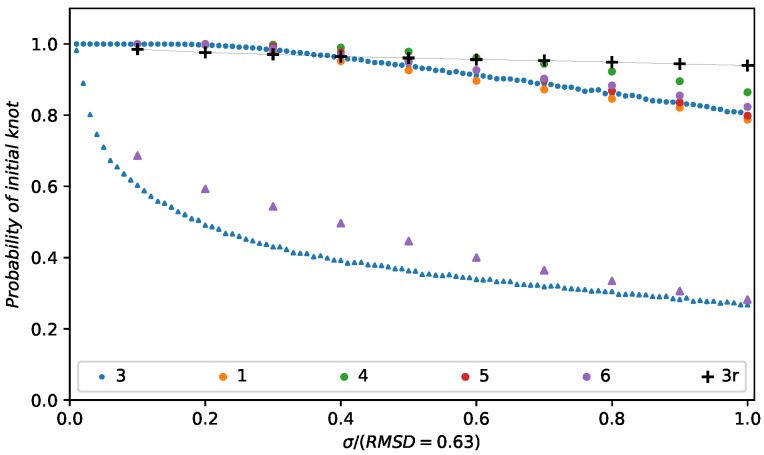
The stability of a knot against random displacements of its nodes. The probability of a change in topology for random displacements of all nodes belonging to the knots highlighted in Figure 4b is shown. The standard deviation of the random displacements increases on going to the right. While the red trefoil knot indicated by blue disks is stable for rather large displacements, the blue trefoil knot indicated by blue triangles is very unstable. Even small displacements are enough to change the topology. A very similar trend can be observed in model 6, which also exhibits a trefoil knot in the blue region (shown as purple triangles). As with model 3, small perturbations are sufficient to change the topology. All the models show a trefoil in the red region, though. The results for these knots are shown as circles. Here, all knots are stable against the displacements. Additionally, displacements of random regions are shown as black crosses with a connecting line as a guide for the eye (3r). These displacements also do not to change the topology.

To quantify the certainty of the topology in the different regions, we checked a knot’s stability against random displacements of their nodes. We measured the probability of an unchanged topology by generating more than 20,000 new models in which all nodes within the knot were randomly displaced following a Gaussian distribution of a certain standard deviation σ. For increasing σ we then measure the fraction of conformations for which the Alexander polynomial remains unchanged. Figure 5 shows the results for model 3 of chromosome 14 in cell 2. This quantitative analysis corroborated our earlier results. The first trefoil that was present in all of the models remains stable in more than 80% of the random conformations tested, for displacements of the order of this structure’s RMSD [48]. This does not hold true for the other trefoil. It vanished with very small changes in the structure. At σ=0.2 RMSD less than 50% of the models have the original topology. The same trend can be observed in the other models. Furthermore, Figure 5 shows the results for all models that exhibit either one or both trefoils and no further knots. While the first trefoil remains stable in all models, the probability of finding the second trefoil decreases rapidly for small displacements in both models that show a knot in that region of the unmodified structure (models 1, 3, 4, 5 and 6). One can see that all models fall into the same respective classes of stable or unstable knots. Other knots that were studied show similar trends. Their behavior ranges from a stability comparable to the first knot to even more unstable characteristics than the second knot. For comparison, the same analysis was done for random regions of the size of the stable knot (29 beads, cell 2, chromosome 14, model 3). Here, the behavior is similar to that of the trefoil, indicating that the formation of knots in the structures by small random displacements is rather unlikely.

## 4. Conclusions

In summary, we have analyzed for the first time the occurrence of knots in experimental structures of chromosomes in the cell nucleus [48]. We find that the (ground-state-like) topological structure of the chromosomes is well defined by a combination of the underlying contact data and model potentials. Although the analysis is necessarily limited by the simplicity of the chromosomal computational model used in the structure calculations, where a 100 kb of nucleosome-wrapped DNA of unknown higher order structure is considered as one bead, we find evidence that at least some knots are likely to be real. This finding is consistent with the fact that plots of contact probability vs sequence separation show a power law dependence that is intermediate between that of a fractal globule and an equilibrium structure.

## Figures and Tables

**Table 1:** Frequency of occurrence of the simplest and thus most common knot types found in the given structures. While the second column shows the raw output of our analysis, the third column gives the numbers after correcting for knots that are only likely to arise from the closure. All knots with termini within 20 beads of the ends of the chain are discarded. Only simple knots are shown as our detection of the knot termini is not reliable for composite knots.

Knot Type	Frequency	Corrected Frequency
Unknot	17.9%	
$3_{1}$	14.8%	12.3%
$4_{1}$	2.4%	1.8%
$5_{1}$	1.3%	1.0%
$5_{2}$	1.6%	1.1%
$3_{1}\#3_{1}$	4.6%	
$3_{1}\#4_{1}$	2.6%	
More complicated	54.8%	
